# Exploratory study of a screening measure for polycystic ovarian syndrome, quality of life assessment, and neuropsychological evaluation

**DOI:** 10.1186/s12905-020-00994-8

**Published:** 2020-06-23

**Authors:** Michael J. Boivin, Farnaz Fatehi, Amy E. Phillips-Chan, Julia R. Richardson, Amanda N. Summers, Steven A. Foley

**Affiliations:** 1 Department of Psychiatry and Neurology & Ophthalmology, Michigan State University, East Lansing, 909 Wilson Road, Rm 327, West Fee Road, East Lansing, MI 48824 USA; 2 Department of Psychology, Indiana Wesleyan University, Marion, Indiana USA; 3 Prowers Medical Center, Lamar, CO USA

**Keywords:** Polycystic ovarian syndrome (PCOS), Quality of life, Fatigue, Depression, Anxiety, Spiritual wellbeing, Neuropsychology, Cognitive performance, Computer assessment

## Abstract

**Background:**

The universally adopted 2018 PCOS medical diagnostic and treatment guidelines for Polycystic Ovarian Syndrome (PCOS) cites the need for a brief screening measure that can be easily administered in the clinical care setting. We evaluate a 12-item questionnaire emphasizing the medical symptoms of PCOS with a group of women with PCOS as well as comparison samples of college women not diagnosed with PCOS.

**Method:**

Of 120 undergraduate psychology women 18 to 41 years of age, 86 screened negative on a 12-item PCOS symptoms inventory. They were compared to a group of PCOS patients diagnosed medically in a manner consistent with the Teede et al. (2018) evidence-based diagnostic guidelines. The screen-positive, screen-negative, and PCOS-confirmed groups were compared on the PCOS Quality-of-Life (QoL) questionnaire, Zung Self-Rating Depression Scale (ZDS), Spielberg State-Trait Anxiety Inventory (STAI), Fatigue Symptom Inventory (FSI), Spiritual well-being and Spiritual Beliefs Inventories, the computerized Automated Neuropsychological Assessment Metric (ANAM) battery, and an experimental tachistoscopic Bilateral Perceptual Asymmetries Letter and Dots Matching Bilateral Field Advantage (BFA) test (to evaluate the effects of early brain androgenization possible from PCOS). For each questionnaire and neuropsychological performance principal outcome, the Linear Mixed Effects (LME) model was employed to evaluate the predictive significance of demographic characteristics and group membership (confirmed cases, screen negative and screen positive cases) for these outcomes.

**Results:**

The PCOS-confirmed women scored more poorly than the screen-negative (reference) and screen-positive groups on all the measures of physical, emotional, social, and spiritual well-being measures. On the ANAM neuropsychological battery, PCOS-confirmed women did more poorly on Sternberg Memory and Stimulus Response throughput measures. They also had slower correct response speed for both the unilateral and bilateral dot- and letter-matching tachistoscopic stimulus presentations. However, the bilateral field advantage throughput performance ratio did not differ among groups, which is a global measure of bilateral versus unilateral brain/behavior asymmetries.

**Conclusion:**

PCOS screening can be a feasible and important part of women’s healthcare. PCOS-confirmed women should receive not only the medical standard of care from the 2018 guidelines, but also comprehensive psychosocial and neurocognitive support to enhance their quality of life.

## Background

Polycystic ovary syndrome (PCOS) is one of the most prevalent endocrinopathies in women of child-bearing age, affecting up to 10% of American women [1]. It is caused primarily by a lack of sensitivity of the body tissue to insulin in response to elevated blood glucose levels, leading to hyperinsulinemia [2]. Women with the genetically-based condition are consequently much more at risk for Type II diabetes mellitus, hypertension, elevated blood lipid and cholesterol levels, and cardiovascular disease [3]. Typically related to these metabolic risk factors is an increased level of testosterone due to decreased sex hormone binding globulin (SHBG) [4]. There is also an increased ratio of Luteinizing Hormone/Follicle Stimulating Hormone (LH/FSH) and increased adrenocorticotrophic hormone (ACTH) [5]. This sometimes leads to obesity, development of greater muscle mass, more body hair (hirsutism), and thinning of scalp hair. Ovulation cycle is often disrupted due to low FSH levels in these women that sometimes causes the emergence of multiple small cysts on the ovaries (hence, the term polycystic) raising concerns about infertility [2].

Böttcher and colleagues (2017) characterized PCOS as a heterogeneous condition, usually observed in women of reproductive age, with such symptoms as infertility, hirsutism, obesity, amenorrhea (irregular menses), insulin resistance, and increased androgen levels [6]. These symptoms can significantly impact body image and overall quality of life (QoL), leading to higher levels of anxiety and depression because these features affect the outside appearance and social norms [7]. Related to these problems of emotional well-being (EWB) is the observation that PCOS is often accompanied by obesity, a perceived lack of adequate family support, and poor satisfaction with sexuality [8]. Socio-economic standing (SES), professional occupation, age at and time from PCOS diagnosis, the presence of acne, body-mass-index (BMI), and perceived infertility status can all be important modifiers of these QoL and EWB effects in women with PCOS [9, 10]. Consequently, a healthy lifestyle of regular exercise and a well-balanced diet can significantly enhance QoL and EWB in women with PCOS [11]. Poor quality of sleep can also be symptomatic of PCOS [12], and an important dimension of QoL for women experiencing emotional difficulty coping with this condition [11]. Kalmbach and colleagues (2017) observed a relationship between nightly sleep disturbance and daily experiences of depression and anxiety [13, 14]. Poor sleep quality may be contributing to the EWB effects of heightened depression that is often observed in women with PCOS.

Because of the potential impact of elevated androgen levels and early brain development in women, a few studies have attempted to explore the relationship between levels of free testosterone (estimated by the free androgen index) and performance on tests of verbal fluency, verbal memory, manual dexterity, and visuospatial working memory in women with and without PCOS [15]. Schattmann and Sheerwin (2007) concluded that PCOS women show poorer results on these cognitive tasks compared to non-PCOS women [15, 16]. They also concluded that the pharmacologic manipulation of free testosterone levels did not have a significant impact on cognitive performance in women with PCOS, although reductions in free T may be beneficial for verbal fluency [16].

Barnard et al., (2007) observed increased MRI brain activity in the parietal lobe for visual-spatial processing tasks in women with PCOS, compared to non-PCOS women [17]. This could be interpreted as less brain/behavior efficiency for such tasks due to the greater degree of neural-network activity for PCOS women for such tasks, perhaps because of the neurodevelopmental effects of brain androgenization presumed to take place with PCOS. These conclusions were supported by a study by Rees et al. (2016) in which they compared cognitive performance as it related to MRI diffusion tensor imaging (DFI) measures between PCOS and non-PCOS women [18]. They found that cognitive performance was poorer in PCOS women and that these deficits were related to MRI DTI white matter microstructure indices, suggesting poorer structural efficiencies. These cognitive and MRI DFI relationships in PCOS women were independent of age, education, and BMI.

In terms of developing a quality of life questionnaire specific to PCOS, the PCOS Quality-of-Life Scale (PCOQ) was developed by researchers at Brigham and Women’s Hospital, Boston, Massachusetts [19]. The present version consists of 26 items representing the four domains of quality of life. In order to develop their health-related quality-of-life questionnaire for women with PCOS, Cronin et al. (1998) started with a pool of 183 potentially relevant items. This pool of items was administered to 100 women medically diagnosed with PCOS. Medical diagnosis consisted of having at least several of the following symptoms: high androgen levels (blood tests), cholesterol or triglyceride levels, insulin levels, irregular menstrual cycles, and cysts in the ovaries confirmed by ultrasound. Symptoms of excessive acne, face and body hair growth, and weight gain were also taken into consideration.

Items among the pool of 183 PCOS-relevant questions endorsed by at least 50% of these women were included in a factor analysis, which resulted in five domains for PCOS quality of life. The cluster of items with the highest factor loading from these five domains comprised the final 26 items for the PCOQ instrument and take about 10 to 15 min to complete. The questionnaire items pertain to emotions (8 items), body hair (5 items), weight (5 items), infertility concerns (4 items), and menstrual problems (5 items). The PCOQ instrument is published in Cronin et al. (1998) as an appendix [19].

In 2018 international PCOS evidence-based medical guidelines were published based on recommendations from the international society-nominated panels which included specialists in pediatrics, endocrinology, gynecology, primary care, reproductive endocrinology, obstetrics, psychiatry, psychology, dietetics, exercise physiology, and public health. This 15-month deliberative process involved 37 societies and organizations covering 71 countries [20–22]. Among many other diagnostic and treatment guidelines were recommendations for screening the impact of this medical condition on a broad range of women’s health issues pertaining to quality of life. The present study attempts to evaluate a shorter 12-item version of the PCOQ adapted by one of the co-authors (SAF) called the Foley Polycystic Ovarian Syndrome Screening Scale (FPCOS). This was adapted as a screening instrument for PCOS and is based on twelve of the PCOQ items pertaining to medical symptoms with the strongest factor loadings in the Cronin et al. (1998) study [19]. The principal study goal is to evaluate the utility of the FPCOS as a screening instrument for clinical practice.

We will do so by comparing women medically diagnosed with PCOS to university students screening higher or lower on the FPCOS. We will compare these three groups on other questionnaires evaluating overall general quality of life (QoL), emotional well-being (EWB) (e.g., depression, anxiety), symptoms of fatigue, social support, and spiritual well-being (SWB). We will also compare our PCOS patients to PCOS screen-positive and screen-negative college women on computerized neuropsychological performance. We hypothesize that women medically diagnosed with PCOS will have significantly poorer QoL compared to the non-PCOS groups, and that this poorer QoL will be related to poorer EWB and SWB. Furthermore, we anticipate that the neuropsychological profile of PCOS patients will be related to their QoL and EWB measures, perhaps differentiating a core brain/behavior symptomology reflective of the neurodevelopmental impact of the endocrinopathy features of this syndrome.

## Methods

### Compliance with ethical standards

This study was approved by the Institutional Review Board (IRB) at Indiana Wesleyan University (IWU). Women participated only after providing informed written consent, and all procedures performed in this study were in accordance with the ethical standards of the institutional and/or national research committee and with the 1964 Helsinki declaration and its later amendments or comparable ethical standards.

### Participants

For this study, 120 women between the ages of 17 to 42 years of age enrolled in a 2nd year psychology course at Indiana Wesleyan University were given the option to participate in our study for academic extra credit. If they agreed to participate by signing a written consent form explaining the study, they completed the Foley Polycystic Ovarian Syndrome (FPCOS) screening scale for symptoms of PCOS. Their score on this instrument determined if they were in the “screen negative” or “screen positive” group in the present study. Screen positive women were offered a subsequent medical appointment that was offered along with a medical follow-up evaluation. IWU women students scoring high on the Foley PCOS screening assessment were individually interviewed by Steven A. Foley (SAF) and recruited into the study through the IWU health center if confirmed to have PCOS following analysis of a blood draw for insulin resistance markers, blood androgen levels, as well as elevated cholesterol and triglyceride level (*N* = 11, PCOS-confirmed group). Eight of the IWU psychology students scored high enough on the Foley PCOS screening questionnaire for medical follow-up but declined to participate and remained in the “screen positive” group in the present study. Five women scored negative on the PCOS screening measure but did not attend their appointments for completing the other study assessments and were not included in this study.

#### Instruments

##### Foley polycystic ovarian syndrome screening scale (FPCOS)

The PCOS Foley Screening Instrument was developed by SAF to assess the medical risk for PCOS. Using the most significant factor loadings for medical items from Cronin et al., (1998) Foley devised a 12-item screening questionnaire for PCOS for use in the medical women’s health clinical setting [19]. At the time, the Cronin et al. (1998) medical PCOS quality of life questionnaire was the most cited instrument in evaluating these domains with PCOS patients [7, 23, 24]. Likewise, at the time of this study, the 2018 International Guidelines for the assessment and management of PCOS had not yet been released, so women’s health practitioners used a variety of medical symptoms to try to screen for and diagnose PCOS in the healthcare setting [20–22].

The FPCOS screening items were selected by SAF as medically strategic in diagnosing the symptomology of PCOS on the basis of an authoritative medical book written by Samuel S. Thatcher (2000) [25]. On a scale from 0 (no history of problems) to 10 (consistent history of problem), so that the higher the score the poorer the health and quality of life self-evaluation by the respondent. The FPCOS has a question dedicated to each of the following 12 items: high cholesterol, high triglycerides, problems with weight loss, cravings for sweets, muscular weakness, excessive body hair, acne problems, father had excessive hair, sudden weight gain, difficulties conceiving children, history of miscarriages, and significant menstruation discomfort.

The rating for all 12 items is totaled and a scored of greater than 40 indicates a significant risk for PCOS, necessitating follow-up medical evaluation if the participant is agreeable. Medical confirmation of PCOS was made with ultrasound imaging evidence of cysts on the ovaries, significantly high levels of low-density lipoprotein (LDL) and high-density lipoprotein (HDL) cholesterol and triglyceride levels from lipid blood panels, along with a high body mass index (BMI) – all of which are risk factors of metabolic syndrome women. These clinical criteria, described in detail by Thatcher (2000), served as diagnostic measures were used by SAF to identify the medically “confirmed PCOS” women in the present study [25]. The FPCOS has only been used by SAF only in his medical practice specializing in women’s health issues and has not been evaluated for sensitivity or specificity as a screening measure. The present study is the first time that it has been evaluated for its utility as a screening instrument for PCOS. The principal PCOS quality of life (QoL) measure is correlated to the FPCOS measure. The PCOS QoL measure is described next.

**The PCOS Quality-of-Life Scale was devised** by researchers at Brigham and Women’s Hospital, Boston, MA and validated with a sample of 100 clinically diagnosed PCOS women [19]. As described above, the present version consists of 26 items provided a composite total PCOS QoL score as an overall item average on a scale from 1 to 10, with a higher score indicating a better QoL.

##### **Zung self-rating depression scale** [26]

Used by our group in a previous women’s health study in this study setting pertaining to breast cancer treatment [27], this is a 20-item self-administered questionnaire that takes only a few minutes to complete. It includes a variety of statements associated with depressed moods and is a helpful tool to assess depression in individuals in a general medical setting [28]. The inventory looks at various symptoms of depression such as insomnia, poor appetite, fatigue, suicidal thoughts, anhedonia, and dysphoria. The 20 items are based on a Likert scale and the four possible responses range from “None or little of the time” to “Most or all of the time.” The higher the subject scores on the Zung scale, the more at risk a respondent is for depression.

**The State-Trait Anxiety Inventory*(STAI)*** is a 40-item measure that looks at both state (in the moment" and trait (chronic) anxiety (Spielberger, 1977) [29]. This questionnaire was used by our group in a previous women’s health study pertaining to breast cancer treatment [27]. This instrument has been used effectively to characterize anxiety in adolescent and adult women with PCOS [30, 31]. For the present analysis, we used the trait anxiety measure.

##### Fatigue symptom inventory (FSI) [32–34]

Originally developed for cancer patients and used by our group in a previous women’s health study pertaining to breast cancer treatment, [27] this is a 14-item self-report measure for measuring the intensity, frequency, and impact of symptoms of fatigue on a woman’s quality of life. Higher scores indicate more fatigue symptoms.

##### Bottomley social support scale (BSS) [35]

This is a seven-item scale originally designed for cancer patients and used by our group in a previous women’s health study pertaining to breast cancer treatmen [27]. This measure was adapted for the present study by removing specific references to “cancer.” It was then used to gauge the social support available to medical patients and the extent to which they utilize available resources in coping with any chronic medical need and its treatment. Each item is rated on a scale between 1 and 5. Women had options to indicate that the item was not applicable or refuse to respond. Total scores range from 7 to 35. Higher scores indicate less perceived social support by our study women.

##### Spiritual beliefs inventory (SBI) [36]

This is a well-validated 15-item questionnaire that is a brief, yet robust measure of the more universal aspects of religious, spiritual and community social support while coping with a life-threatening illness as well as the subsequent quality of life (QoL) issues, particularly in the context of cancer care [37]. Higher scores indicate a stronger spiritual QoL. This measure of spirituality was used previously by our group in a study pertaining to breast cancer treatment [27].

**Automated Neuropsychological Assessment Metric (ANAM*)*** [38] is a computerized neuropsychological assessment developed by Dr. Joe Bleiberg at the National Rehabilitation Hospital in Washington, D.C. for a PC laptop in the hospital or clinic setting. This assessment is used in human performance factor studies (e.g., neuropsychological effects of fatigue, chronic stress, sleeplessness, toxic exposure and was developed by researchers at the Walter Reed Medical Center [38–40]. The framework for this assessment is derived from the Halstead-Reitan Neuropsychological Assessment Battery and the Wechsler Adult scales for both intelligence (WAIS) and memory (WMS). Measures such as the Tower of Hanoi Task, Symbolic Logical Relations Test), Sternberg encoding and memory, Sternberg running memory, spatial processing (sequential and simultaneous), and a running memory continuous performance task – provided measures for the neurocognitive domains of executive functioning, problem solving, attention, memory and learning, and processing speed for all of these domains. Although speed, error, and variability are provided for each test in the ANAM, we used the throughput measure (speed by accuracy) for each test as our principal outcome in the present analysis. This was the principal neuropsychological assessment battery used by our group in a previous women’s health study pertaining to breast cancer treatment, where we were able to observe its applicability and validity in that women’s health context [27]. The higher the score, the better the performance.

**Fig. 1:**
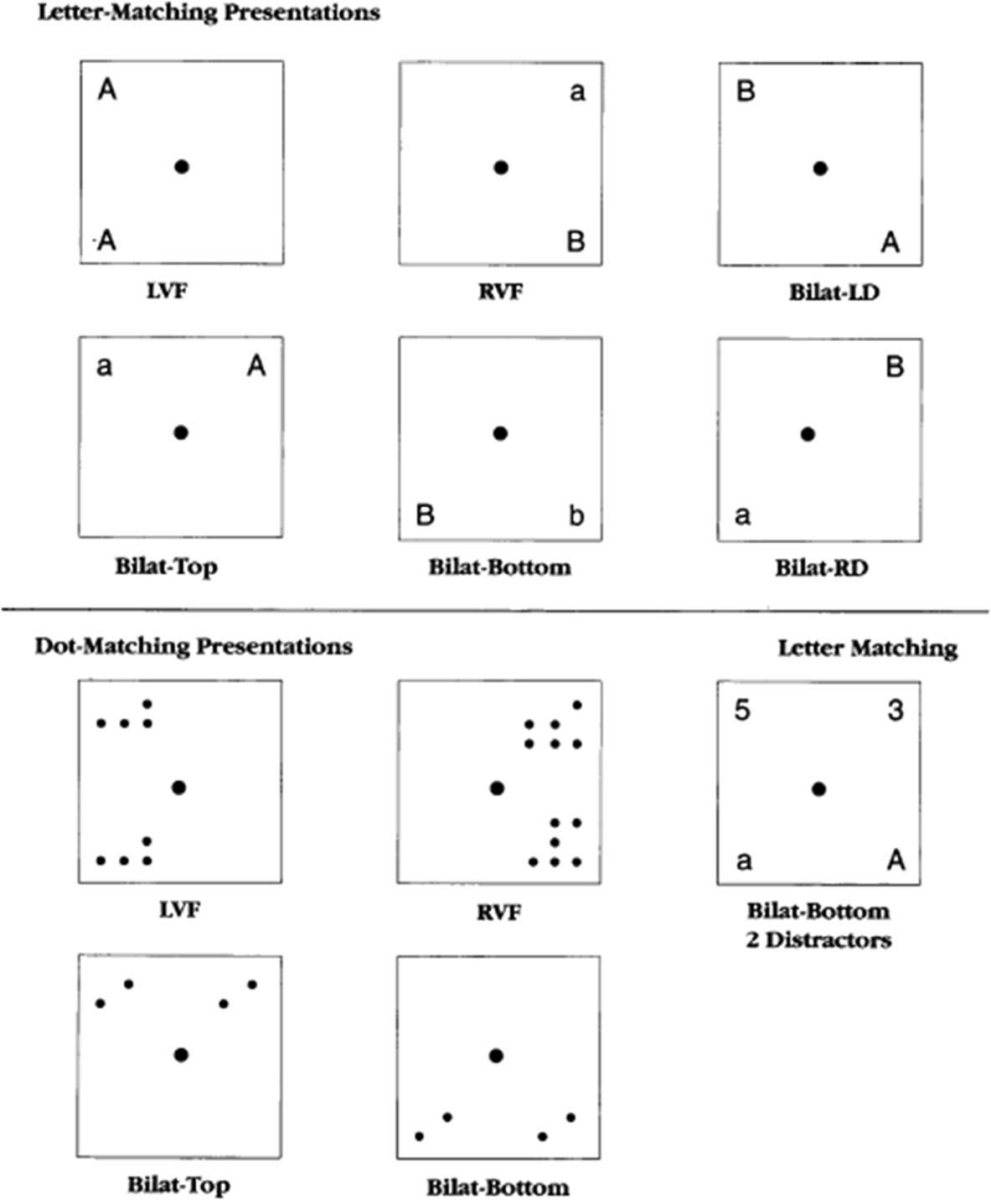
Presentation positions used for the letter-matching presentations (upper – part 1 of test) and the dot-matching presentations (lower – part 2 of test) for the computerized bilateral field advantage (BFA) dot and letter matching tachistoscopic task. These are labeled by left-visual field (LVF), right-visual field (RVF), bilateral left diagonal (Bilat-LD), bilateral top (Bilat-Top), bilateral bottom (bilat-Bottom), and bilateral right diagonal (bilat-RD)

**Bilateral Field Advantage (BFA) task of interhemispheric brain integration is** a computerized assessment [41, 42]. Boivin and colleagues have previously used this assessment along with measures of spiritual wellbeing with young adults in the university setting [43]. In previous experimental studies with such students, this test proved sensitive in evaluating the efficiency of right and left hemispheres to process simple visual information, using both a dot (visual-spatial processing) or a letter-matching (verbal processing) task presently tachistoscopically (very rapidly). This is the first time that this experimental neurocognitive performance measure has been used in women’s health research to characterize bilateral field advantage (right or left brain dominant) in neurocognitive performance. We administered a computer-based visual-perceptual asymmetries task (see Fig. 1)**.** The stimuli were generated by computer from its standard character set and briefly displayed tachistoscopically on a 17 by 11-in. computer monitor at a viewing distance of about 15-in.. A letter pair from the grouping, for example, “AaBb”, was presented for each trial (see Fig. 1) [43]. Anywhere from 0 to 3 distractor digits could be presented with the matching or non-matching letter pairs (e.g., Aa, AB or Ab, AA). The letters comprising the pair could also be presented across four different visual fields (left, right, bilateral-top, bilateral-bottom) Fig. 1) [43]. If one member of each letter pair was presented in the left visual field and the other member in the right visual field, then this was considered a bilateral field presentation. For bilateral field presentations, both letters could appear either in the upper portion of the screen (Bilateral Top) or bottom portion (Bilateral Bottom). Letter pairs could also be both presented in the same visual field (left or right); either both in the upper, bottom, or diagonal positions (Fig. 1) [43]. Each condition was presented in a total of five trials during the session, which lasted about 30 min.

### Statistical analyses

Descriptive statistics were obtained for each PCOS risk group (confirmed cases, screen negative and screen positive cases). The chi-square and analysis of variance were used to compare groups at intake on age, education and income. Then, outcomes at intake listed in Table 1 were compared among 3 groups while adjusting for the demographic characteristics to determine the effects of cancer diagnosis. For each questionnaire and neuropsychological performance principal outcome, the Linear Mixed Effects (LME) model was employed to evaluate the predictive significance of demographic characteristics and group membership (confirmed cases, screen negative and screen positive cases) for these outcomes. Unfortunately, our PCOS medically confirmed sample size (*N* = 11) from our base sample of 120 women is too small for us to compute a sensitivity or specificity analysis for our FPCOS screening measure, assuming a disease prevalence of 10% or less and a power of 0.80 at a 5% significance level [44]. Our statistical analyses could only be correlational in nature, as a preliminary evaluation of the possible utility of this screening tool in a university population of young women.

**Table 1:** Descriptive statistics for the quality of life and emotional wellbeing questionnaire measures for the PCOS screen negative, PCOS screen positive, and PCOS medically confirmed groups. (group mean compared to reference group: *p < 0.05; **p < 0.02; ***p < 0.001)

	Screen Negative (reference group)				Screen Positive				PCOS Confirmed			
	N	Mean	Std. Deviation	Std. Error	N	Mean	Std. Deviation	Std. Error	N	Mean	Std. Deviation	Std. Error
Age	74	18.55	.89	.10	25	19.00	2.449	.49	11	29.09***	6.68	2.01
Years of Formal Education Post-HS	74	3.43	.66	.07	25	3.56	.58	.12	11	4.45***	1.36	.41
Foley PCOS Screening Scale	80	24.85	7.70	.86	30	47.30***	7.50	1.40	12	60.81***	14.50	4.20
Zung Depression Total	74	32.50	6.65	.77	25	35.56	7.07	1.41	10	46.20***	12.66	4.05
State Trait Anxiety Inventory Total	74	35.86	10.56	1.22	25	40.36	9.89	1.97	10	46.10*	15.61	4.93
PCOS Quality of Life Total	73	12.67	3.54	.41	25	13.72	3.29	.66	10	8.80**	5.02	1.59
Spiritual Wellbeing Total	73	42.90	6.52	.76	25	41.16	4.93	.98	10	28.00***	11.96	3.78
Spiritual Belief Inventory Total	74	40.34	5.31	.61	25	39.28	4.73	.94	10	32.30***	10.74	3.39
Fatigue Symptoms Inventory Total	74	40.38	21.08	2.45	25	52.44*	18.28	3.65	10	60.40*	23.50	7.43
Bottomley Social Support Scale Total	74	11.20	4.43	.51	25	12.24	4.45	.89	10	17.80***	6.08	1.92

## Results

**Fig. 2:**
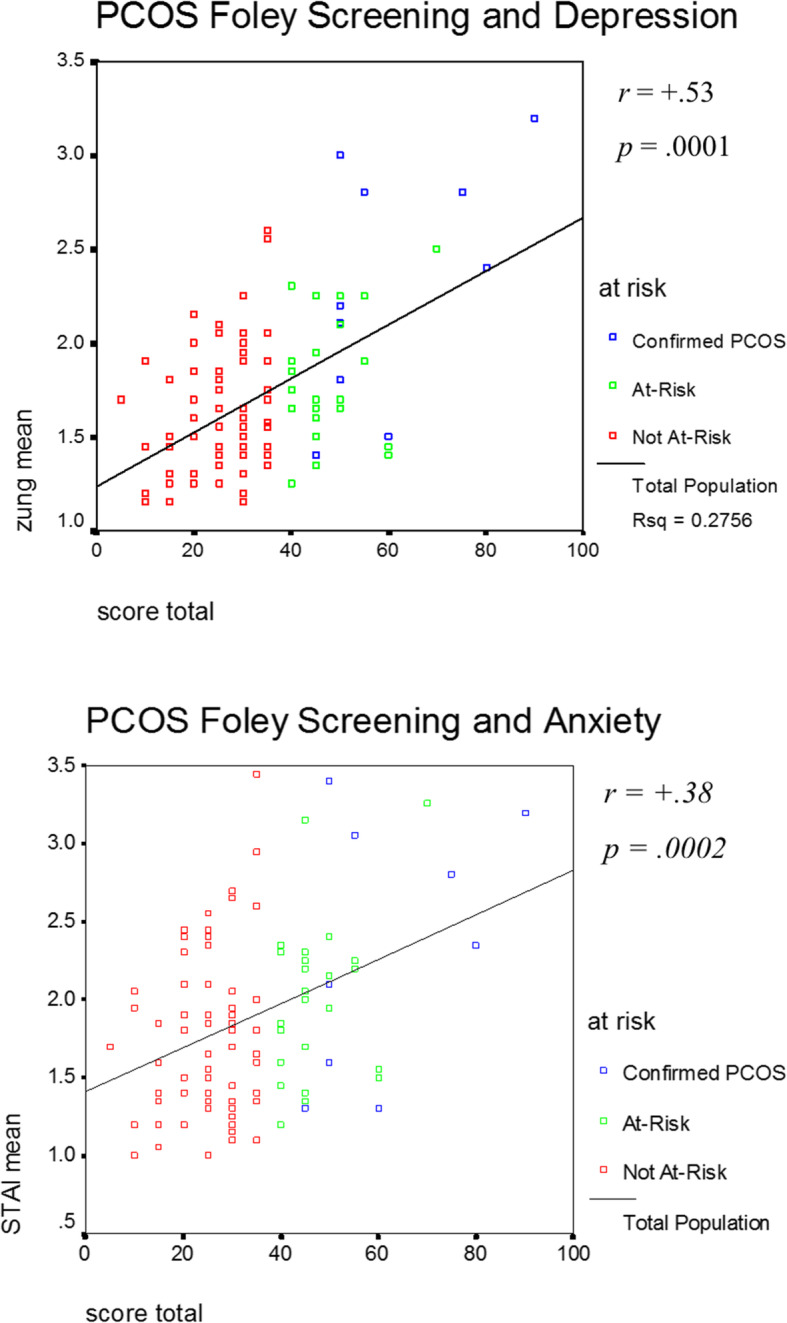
Scatterplot and least-squares fit line for depicting the relationship between Zung Depression Scale and the Foley PCOS screening measure total symptom score (upper graph), and the State-Trait Anxiety Inventory (STAI) total and the Foley PCOS screening total number of symptoms (lower graph)

The PCOS-confirmed women were significantly older than the screen positive or negative comparison groups (*p* < 0.001) and had more years of formal education post high school (*p* < 0.05) (Table 1). The PCOS-confirmed women (*N* = 10) had significantly poorer emotional well-being and quality of life than the screen-positive (*N* = 25) and screen-negative (*N* = 74) groups of women. These significant between-group differences included the following: Zung Depression Scale (total score), F (2,106) = 15.72, *p* < .001; State-Trait Anxiety Inventory (STAI: total score), F (2,106) = 4.72, *p* = .01; Quality-of-Life Scale, F (2,106) = 41.46, *p* < .001; Fatigue Symptom Inventory, F (2,106) = 6.22, *p* = .003; Bottomley Social Support Scale, F (2,106) = 10.00, *p* < .001; and the Spiritual Beliefs Inventory (total score), F (2,106) = 4.57, *p* = .01 (Table 1). Between-group differences on the Zung depression and STAI anxiety scales are depicted as box plots in Fig. 2. The relationships between these depression and anxiety scores, and the PCOS screening inventory is significant for all the study women across all three groups along with their respective correlation coefficients (*p* < 0.001).

On the Automated Neuropsychological Assessment Metric (ANAM), the PCOS patients did significantly more poorly on the Sternberg Memory Recall Test Throughput measure (both accuracy and speed), F (2,104) = 5.84, *p* = .004 (Table 2). They also did more poorly on the Symbolic Relations Test throughput (*p* < 0.01). There were no significant differences among the three groups on any of the other ANAM performance measures (Table 2).

**Table 2:** Computerized Automated Neuropsychological Assessment Metric (ANAM) throughput (accuracy by speed) performance measures for PCOS screen negative, PCOS screen positive, and PCOS medically confirmed groups. (group mean compared to reference group: *p<0.05; **p<0.02; ***p<0.001)

	Screen Negative (reference group)				Screen Positive				PCOS Confirmed			
	N	Mean	Std. Deviation	Std. Error	N	Mean	Std. Deviation	Std. Error	N	Mean	Std. Deviation	Std. Error
Tower Hanoi Puzzle Throughput	73	3.41	1.19	.14	25	3.72	1.51	.30	9	2.00**	.50	.16
Symbolic Logical Relations Throughput	73	213.19	43.47	5.08	25	225.97	32.69	6.53	9	168.01**	31.84	10.61
Math Processing Throughput	73	20.03	8.24	.96	25	22.20	6.30	1.26	9	21.67	6.18	2.06
Continuous Attention Monitoring Throughput	73	37.85	11.67	1.36	25	39.37	12.53	2.50	9	34.42	12.60	4.20
Spatial Processing Throughput	73	27.05	7.10	.83	25	26.00	5.91	1.18	9	26.25	8.64	2.88
Sternberg Memory Recall Throughput	73	78.35	18.95	2.21	25	82.29	14.86	2.97	9	59.17**	12.61	4.20
Sternberg Running Memory Throughput	73	105.12	25.40	2.97	25	106.78	23.27	4.65	9	99.55	17.88	5.96

On the Bilateral Field Advantage (BFA) test, which is a tachistoscopic dot and letter matching task (Fig. 1), both the PCOS-confirmed and PCOS screen-positive groups had a stronger unilateral correct response time performance to the visual field for the brain hemisphere dominant for that task (dot matching: left visual field/right hemisphere versus letter matching: right visual field/ left hemisphere). Non-at-risk women had faster correct response times as well as more correct responses for dots and letters presented bilaterally, compared to the PCOS-confirmed women (Table 3). The same between-group differences were seen for the accuracy measures for our study participants on the BFA test. However, the final bilateral field advantage ratio (bilateral throughput divided by unilateral throughput) among the groups did not significantly differ (Table 3, bottom). Among all women in the present study, the higher the score on the PCOS screening instrument, the greater the tendency towards a unilateral as opposed to bilateral field advantage on the BFA dot matching test (*p* = 0.0002).

**Table 3:** Computerized bilateral field advantage (BFA) dot and letter matching tachistoscopic task mean on correct responses (msec): descriptive statistics for PCOS screen negative, PCOS screen positive, and PCOS medically confirmed groups. (group mean compared to reference group: *p < 0.05; **p < 0.02; ***p < 0.001)

	Screen Negative (reference group)				Screen Positive				PCOS Confirmed			
	N	Mean	Std. Deviation	Std. Error	N	Mean	Std. Deviation	Std. Error	N	Mean	Std. Deviation	Std. Error
Match dot unilateral mean	70	769.21	115.42	13.79	24	760.58	128.18	26.16	10	842.00*	137.83	43.58
Match dot bilateral mean	70	748.10	106.84	12.77	24	700.37	172.55	35.22	10	839.70*	120.33	38.05
No match dot unilateral mean	70	750.64	120.42	14.39	24	767.00	113.30	23.12	10	865.20*	112.50	35.57
No match dot bilateral mean	70	722.17	156.98	18.76	24	758.91	104.85	21.40	10	841.30*	120.10	37.97
Match letter unilateral mean	62	764.40	160.65	20.40	21	757.00	144.39	31.51	11	889.20*	147.90	44.59
Match letter bilateral mean	61	727.13	124.34	15.92	21	724.80	129.86	28.33	11	839.18*	137.76	41.53
No match letter unilateral mean	61	789.36	124.21	15.90	21	789.19	125.02	27.28	11	905.09*	147.15	44.36
No match letter bilateral mean	61	758.14	127.32	16.30	21	773.80	92.45	20.17	11	880.36**	150.85	45.48
No match letter BFA mean	61	32.02	50.29	6.44	21	15.38	54.78	11.95	11	24.73	44.724	13.48
Overall BFA for mean reaction time (throughput bilateral/unilateral ratio)	73	24.85	38.45	4.50	24	22.75	22.72	4.63	11	26.91	29.895	9.01

Our final analysis consisted of a multiple regression analysis evaluation. Using a stepwise approach for the most significant predictors of differences among the study groups from the present assessment domains, we incorporated anxiety, depression, and BFA unilateral field advantage as the optimal predictive model for overall number of symptoms on the PCOS Screening Instrument (*r* = 0.634, *p* = 0.009).

## Discussion

Our findings document that women medically confirmed with PCOS are at risk for greater anxiety and depression and poorer quality of life in terms of social support and spiritual wellbeing. They are also subject to poorer neuropsychological performance on memory tasks emphasizing efficiency and speed of processing, as well as a symbolic relations test of executive function. PCOS-confirmed women also had overall slower performance on a dot and letter matching test of visual perceptual asymmetries for unilateral as opposed to bilateral tachistoscopic stimulus presentations. They did not, however, display a greater bilateral field advantage on throughput measures, when compared to the PCOS screen-negative (reference group) and screen-positive women.

Given the small number of PCOS screen-positive women who were then medically confirmed for PCOS in the present study, our study findings can only be considered preliminary at best. However, they reflect a comprehensive evaluation of the emotional, social, and spiritual wellbeing of these women, and ways in which their quality of life is related to their neuropsychological performance. Furthermore, the present findings provide preliminary evidence for the importance of screening women for symptoms of PCOS as part of their general medical care. This is not only for the sake of further clinical evaluation when indicated, but also because these symptoms are related to quality of life and neurocognitive function even in the absence of a full-blown endocrinopathy such as PCOS.

The present study did not evaluate the direct pathophysiological relationship between PCOS symptoms and our emotional, social, and spiritual wellbeing and neurocognitive performance measures in our screen positive women. Furthermore, the confirmed PCOS cases in our study were under medical care and treatment by a coauthor (SAF) in this study. As such, we cannot conclude a direct causal pathway between specific hormonal or physiological features of PCOS and quality of life or neuropsychological function (e.g., unilateral/bilateral visual processing propensities). Our exploratory observational data can only suggest further directions for comprehensive evaluative research in PCOS in terms of quality of life and neurocognitive function for women with these symptoms. Our present findings also suggest important outcome domains to monitor in response to treatment for PCOS, and the need for psychosocial support as part of that treatment package.

Amiri and colleagues recently assessed the association between clinical and biochemical characteristics and QoL domains (psychosocial-emotional, fertility, sexual function, and obesity-menstrual) in women with PCOS [45]. As was the case in our study, they used health-related quality-of-life questionnaire for PCOS patients, showing a significant relationship between QoL and such hormonal factors as Dehydroepiandrosterone sulfate (DHEAS; an androgen found in men and women). They also observed that QoL was related to such metabolic biomarkers as triglycerides, total cholesterol, LDL and HDL cholesterol, and HOMA-IR (Homeostatic Model Assessment of Insulin Resistance). These hormonal and metabolic mechanisms would seem to mediate the kind of relationships we observed between QoL, EWB, SWB, and even neurocognitive performance with such PCOS clinical symptoms as obesity, infertility and hirsutism. They concluded that clinicians should regularly assess the clinical and psychosocial dimensions of PCOS as well as biochemical aspects.

The greatest limitation of the present study was the small sample size of medically confirmed PCOS women, who served as a reference group in the exploratory evaluation of our screening measure. Furthermore, our PCOS participants were diagnosed before the 2018 evidence-based guidelines published and universally adapted for PCOS [20–22]. The number of emotional wellbeing and quality-of-life questionnaire measures, along with the performance-based computerized cognitive performance assessment, resulted in many cross-sectional statistical comparisons among PCOS groups identified by the screening measure. Therefore, our statistical findings can only be considered exploratory in nature. As such, the present findings are preliminary and observational.

Despite these limitations, our findings do confirm our hypotheses based on other QoL research with PCOS. One of the hypotheses was that higher scores on the PCOS screening instrument is positively correlated with higher levels of anxiety and depression [46, 47]. These aspects of poor emotional wellbeing may also be related to poor self-esteem in women struggling with these symptoms in the absence of a medical diagnosis for PCOS (screen positive women) [48, 49]. Another hypothesis was that higher scores on the surveys will be related to poorer quality of life in terms of fatigue, stress, social well-being, and low social support [24, 50]. This was the case in our present between-group comparisons between PCOS screen-positive and screen-negative women. For example, Ghazeeri and colleagues found that the latency of androgens and its contribution to psychiatric illnesses in women with PCOS could be a major factor for the development of psychiatric symptoms, rather than the hyperandrogenic levels by itself [51].

At the onset of the present study, we considered the possibility that there will be higher BFA testing scores related to a greater tendency towards unilateral as opposed to bilateral field advantage in the neuropsychological visual processing test in women diagnosed with PCOS. This could potentially be due to the risk of a greater degree of androgenization during critical periods of brain/behavior development in women with this condition. Although we do not have the hormonal profile and treatment history for these women to establish this relationship more conclusively, our preliminary findings seem to support this possibility. However, Soleman et al. (2016) in their study findings did not support the view that women with PCOS display a more masculine cognitive profile due to hyperandrogenism [52]. Overall, these findings suggested that any impairments were subtle and were unlikely to affect daily functioning. Clearly more work needs to be done, especially in women who screen positive for this condition and who are possibly in need of a more comprehensive endocrinology follow-up and evaluation.

For these women, therefore, in addition to a careful medical follow-up based on a PCOS screen positive evaluation as part of their medical care, we propose the kind of psychosocial and neurocognitive screening evaluation used in the present exploratory study. These can also help guide the need to support for enhancing a woman’s QoL, be it emotional, social, or spiritual. Also, repeated evaluations of the patients’ QoL and coping mechanisms over a long period of time could facilitate early diagnosis of psychological needs or concerns. Hence, therapy and/or support groups for women with PCOS should be made readily available as a standard of care for this condition [46]. Neuropsychological evaluation should also be provided [15, 17].

Smyka and colleagues identified PCOS as one of the most common endocrinopathies of the reproductive age [11]. Addressing lifestyle factors including a diet and physical activity is the most effective way to improve carbohydrate metabolism and achieve weight loss goals, which reactivates regular ovulation and facilitates getting pregnant. Both these lifestyle factors are extremely effective in treating the quality of life problems resulting from PCOS. Patients should be reminded of the crucial role that physical activity plays in augmenting the effects of a well-balanced diet. All of these health practices will also enhance the QoL of women with PCOS, in response to the kinds of needs documented in the present study [11].

A final important QoL concern for women with symptoms of PCOS pertains to their emotional wellbeing as it relates to their self-image and their sexuality. Amiri and colleagues found no evidence of a relationship between low scores for any of their sexual domains evaluated and low serum total and free testosterone levels [53]. However, they did find significant relationships between the low sexual function of PCOS women, and problems with infertility and alopecia. Therefore, the burden of PCOS and sexual dysfunction suggested the need for further attention to this this aspect of QoL, especially PCOS women affected by infertility concerns [54].

## Conclusion

This research has indicated that confirmed PCOS participants scored significantly more poorly on various quality of life measures of physical, emotional, social, and spiritual wellbeing. They also had more difficulty in terms of processing speed and efficiency on select memory, executive function, and dot and letter matching visual processing bilateral field advantage tasks. Scores on the PCOS screening measure used in the present study to differentiate screen-positive to screen-negative women were significantly related to depression, anxiety, and spiritual wellbeing. These were also positively related to dot and letter matching processing speed when presented unilaterally (visual field for one side of the brain only) to our study women.

These findings support the proposal that it might be important to screen for the emotional wellbeing, quality-of-life, and cognitive performance issues often associated with associated with PCOS. Our screening tool is short (12 items) for ease of use in the clinical healthcare setting. Yet our screening questionnaire is sensitive enough to potentially be helpful for medical office visits as part of a standard of evaluative care in women’s health. Those screening positive could then be clinically evaluated for elevated insulin and/or androgen levels associated with PCOS may affect neuropsychological performance and important indicators of quality of life. However, these are only observational findings and preliminary at best. They do support the need for a more comprehensive evaluation of women screening positive for PCOS in terms of their endocrinology, psychosocial, and neuropsychological profiles. Also, screening for PCOS symptoms, quality of life, and neuropsychological performance assessments should become an important part of a more comprehensive general standard of care for women’s health.

## Data Availability

The data that were used in the analyses for the manuscript are not publicly available. They could however be availed upon reasonable request by writing an email to the corresponding author. The Foley PCOS screening measure is available upon request from the author of this instrument, Dr. Steven A. Foley, Prowers Medical Center, 401 Kendall Dr., Lamar, Colorado 81052. Phone: 719 336–3179; FAX: 719 336–7228.

## Abbreviations

ACTH: Adrenocorticotrophic Hormone
ANAM: Automated Neuropsychological Assessment Metric
BFA: Bilateral field advantage
BMI: Body mass index
BSS: Bottomley Social Support scale
DTI: Diffusion tensor imaging
EWB: Emotional wellbeing
FPCOS: Foley Polycyctic Ovarian Syndrome Screening scale
FSH: Follicle Stimulating Hormone
FSI: Fatigue Symptoms Inventory
HDL: High density lipoprotein
IRB: Institutional review board
IWU: Indiana Wesleyan University
LDL: Low density lipoprotein
LH: Luteinizing hormone
LME: Linear mixed effects
LVF: Left visual field
MRI: Magnetic resonance imaging
P: Statistical probability
PCOS: Polycystic Ovarian Syndrome
QoL: Quality of life
RVF: Right visual field
SBI: Spiritual Beliefs Inventory
SES: Socio-economic status
SHBG: Sex hormone binding globulin
STAI: State-Trait Anxiety Inventory
SWB: Spiritual Wellbeing Scale
ZDS: Zung Depression Scale

## References

[1] Sirmans SM, Pate KA (2013). Epidemiology, diagnosis, and management of polycystic ovary syndrome. Clin Epidemiol.

[2] Goodman NF (2015). American Association of Clinical Endocrinologists, American College of Endocrinology, and androgen excess and Pcos society disease state clinical review: guide to the best practices in the evaluation and treatment of polycystic ovary syndrome - part 2. Endocr Pract.

[3] Kelestimur F (2006). Prevalence of polycystic ovarian changes and polycystic ovary syndrome in premenopausal women with treated type 2 diabetes mellitus. Fertil Steril.

[4] Hernandez MI (2017). Hyperandrogenism in adolescent girls: relationship with the somatotrophic axis. J Pediatr Endocrinol Metab.

[5] Genazzani AD (2010). Metformin administration restores allopregnanolone response to adrenocorticotropic hormone (ACTH) stimulation in overweight hyperinsulinemic patients with PCOS. Gynecol Endocrinol.

[6] Bottcher B (2018). Health-related quality of life in patients with polycystic ovary syndrome: validation of the German PCOSQ-G. Arch Gynecol Obstet.

[7] Barnard L (2007). Quality of life and psychological well being in polycystic ovary syndrome. Hum Reprod.

[8] Stapinska-Syniec A (2018). Depression, sexual satisfaction, and other psychological issues in women with polycystic ovary syndrome. Gynecol Endocrinol.

[9] Li Y (2011). Polycystic ovary syndrome is associated with negatively variable impacts on domains of health-related quality of life: evidence from a meta-analysis. Fertil Steril.

[10] Rzonca E (2018). Determinants of Quality of Life and Satisfaction with Life in Women with Polycystic Ovary Syndrome. Int J Environ Res Public Health.

[11] Smyka M, Grzechocinska B, Wielgos M (2018). The role of lifestyle changes in the treatment of polycystic ovary syndrome. Neuro Endocrinol Lett.

[12] Fernandez RC (2018). Sleep disturbances in women with polycystic ovary syndrome: prevalence, pathophysiology, impact and management strategies. Nat Sci Sleep.

[13] Kalmbach DA (2017). Sleep Disturbance and Short Sleep as Risk Factors for Depression and Perceived Medical Errors in First-Year Residents. Sleep.

[14] Kalmbach DA (2017). Reciprocal dynamics between self-rated sleep and symptoms of depression and anxiety in young adult women: a 14-day diary study. Sleep Med.

[15] Schattmann L, Sherwin BB (2007). Testosterone levels and cognitive functioning in women with polycystic ovary syndrome and in healthy young women. Horm Behav.

[16] Schattmann L, Sherwin BB (2007). Effects of the pharmacologic manipulation of testosterone on cognitive functioning in women with polycystic ovary syndrome: a randomized, placebo-controlled treatment study. Horm Behav.

[17] Barnard L (2007). Cognitive functioning in polycystic ovary syndrome. Psychoneuroendocrinology.

[18] Rees DA (2016). White matter microstructure and cognitive function in young women with polycystic ovary syndrome. J Clin Endocrinol Metab.

[19] Cronin L (1998). Development of a health-related quality-of-life questionnaire (PCOSQ) for women with polycystic ovary syndrome (PCOS). J Clin Endocrinol Metab.

[20] Teede HJ (2018). Recommendations from the international evidence-based guideline for the assessment and management of polycystic ovary syndrome. Hum Reprod.

[21] Teede HJ (2018). Recommendations from the international evidence-based guideline for the assessment and management of polycystic ovary syndrome. Fertil Steril.

[22] Teede HJ (2018). Recommendations from the international evidence-based guideline for the assessment and management of polycystic ovary syndrome. Clin Endocrinol.

[23] Bazarganipour F (2013). Predictive factors of health-related quality of life in patients with polycystic ovary syndrome: a structural equation modeling approach. Fertil Steril.

[24] Bazarganipour F (2014). Health-related quality of life in patients with polycystic ovary syndrome (PCOS): a model-based study of predictive factors. J Sex Med.

[25] Thatcher SS (2000). PCOS: the Hidden Epidemic.

[26] Zung WW (1990). The role of rating scales in the identification and management of the depressed patient in the primary care setting. J Clin Psychiatry.

[27] Boivin MJ (2020). Preliminary study on the effects of treatment for breast cancer: immunological markers as they relate to quality of life and neuropsychological performance. BMC Womens Health.

[28] Zung WW, Broadhead WE, Roth ME (1993). Prevalence of depressive symptoms in primary care. J Fam Pract.

[29] Spielberger CD, Vagg PR (1984). Psychometric properties of the STAI: a reply to Ramanaiah, Franzen, and Schill. J Pers Assess.

[30] Laggari V (2009). Anxiety and depression in adolescents with polycystic ovary syndrome and Mayer-Rokitansky-Kuster-Hauser syndrome. J Psychosom Obstet Gynaecol.

[31] Livadas S (2011). Anxiety is associated with hormonal and metabolic profile in women with polycystic ovarian syndrome. Clin Endocrinol.

[32] Hann DM, Denniston MM, Baker F (2000). Measurement of fatigue in cancer patients: further validation of the fatigue symptom inventory. Qual Life Res.

[33] Hann DM (1998). Measurement of fatigue in cancer patients: development and validation of the fatigue symptom inventory. Qual Life Res.

[34] Stein KD (1998). A multidimensional measure of fatigue for use with cancer patients. Cancer Pract.

[35] Bottomley A (1995). The development of the Bottomley Cancer social support scale. Eur J Cancer Care (Engl).

[36] Holland JC (1998). A brief spiritual beliefs inventory for use in quality of life research in life-threatening illness. Psychooncology.

[37] Holland JC (1999). The role of religious and spiritual beliefs in coping with malignant melanoma. Psychooncology.

[38] Reeves DL (2007). ANAM genogram: historical perspectives, description, and current endeavors. Arch Clin Neuropsychol.

[39] Cernich A (2007). Automated neuropsychological assessment metrics sports medicine battery. Arch Clin Neuropsychol.

[40] Roebuck-Spencer T (2007). Assessing change with the automated neuropsychological assessment metrics (ANAM): issues and challenges. Arch Clin Neuropsychol.

[41] Ludwig TE, Jeeves MA (1996). Maximizing the bilateral field advantage on verbal and nonverbal matching tasks. Cortex.

[42] Ludwig TE (1993). The bilateral field advantage on a letter-matching task. Cortex.

[43] Ash CA (1996). Unilateral and bilateral brain hemispheric advantage on visual matching tasks and their relationship to styles of religiosity. J Psychol Theol.

[44] Bujang MA, Adnan TH (2016). Requirements for minimum sample size for sensitivity and specificity analysis. J Clin Diagn Res.

[45] Amiri M (2019). The relationship between clinical and biochemical characteristics and quality of life in patients with polycystic ovary syndrome. Clin Endocrinol.

[46] Cooney LG, Dokras A (2017). Depression and anxiety in polycystic ovary syndrome: etiology and treatment. Curr Psychiatry Rep.

[47] Cooney LG (2017). High prevalence of moderate and severe depressive and anxiety symptoms in polycystic ovary syndrome: a systematic review and meta-analysis. Hum Reprod.

[48] Annagur BB, Tazegul A, Akbaba N (2014). Body image, self-esteem and depressive symptomatology in women with polycystic ovary syndrome. Noro Psikiyatr Ars.

[49] Bazarganipour F (2013). Body image satisfaction and self-esteem status among the patients with polycystic ovary syndrome. Iran J Reprod Med.

[50] Coffey S, Mason H (2003). The effect of polycystic ovary syndrome on health-related quality of life. Gynecol Endocrinol.

[51] Ghazeeri G (2013). Anxiety, cognitive, and depressive assessment in adolescents with polycystic ovarian syndrome: a pilot study. J Pediatr Adolesc Gynecol.

[52] Soleman RS (2016). Does polycystic ovary syndrome affect cognition? A functional magnetic resonance imaging study exploring working memory. Fertil Steril.

[53] Nasiri Amiri F (2018). Sexual function in women with polycystic ovary syndrome and their hormonal and clinical correlations. Int J Impot Res.

[54] Nasiri Amiri F (2014). The experience of women affected by polycystic ovary syndrome: a qualitative study from Iran. Int J Endocrinol Metab.

